# Dynamic performance–Energy tradeoff consolidation with contention-aware resource provisioning in containerized clouds

**DOI:** 10.1371/journal.pone.0261856

**Published:** 2022-01-20

**Authors:** Rewer M. Canosa-Reyes, Andrei Tchernykh, Jorge M. Cortés-Mendoza, Bernardo Pulido-Gaytan, Raúl Rivera-Rodriguez, Jose E. Lozano-Rizk, Eduardo R. Concepción-Morales, Harold Enrique Castro Barrera, Carlos J. Barrios-Hernandez, Favio Medrano-Jaimes, Arutyun Avetisyan, Mikhail Babenko, Alexander Yu. Drozdov

**Affiliations:** 1 Computer Science Department, CICESE Research Center, Ensenada, BC, Mexico; 2 School of Electronic Engineering and Computer Science, South Ural State University, Chelyabinsk, Russia; 3 Control/Management and Applied Mathematics, Ivannikov Institute for System Programming, Moscow, Russia; 4 Information Systems Department, Metropolitan University (UMET), Quito, Ecuador; 5 Computing and Systems Department, University of Los Andes, Bogotá, Colombia; 6 School of Systems Engineering and Informatics, Universidad Industrial de Santander (UIS), Bucaramanga, SA, Colombia; 7 North-Caucasus Center for Mathematical Research, North-Caucasus Federal University, Stavropol, Russia; 8 Sirius University of Science and Technology, Sochi, Russia; 9 Moscow Institute of Physics and Technology, Moscow, Russia; University of Pisa, ITALY

## Abstract

Containers have emerged as a more portable and efficient solution than virtual machines for cloud infrastructure providing both a flexible way to build and deploy applications. The quality of service, security, performance, energy consumption, among others, are essential aspects of their deployment, management, and orchestration. Inappropriate resource allocation can lead to resource contention, entailing reduced performance, poor energy efficiency, and other potentially damaging effects. In this paper, we present a set of online job allocation strategies to optimize quality of service, energy savings, and completion time, considering contention for shared on-chip resources. We consider the job allocation as the multilevel dynamic bin-packing problem that provides a lightweight runtime solution that minimizes contention and energy consumption while maximizing utilization. The proposed strategies are based on two and three levels of scheduling policies with container selection, capacity distribution, and contention-aware allocation. The energy model considers joint execution of applications of different types on shared resources generalized by the job concentration paradigm. We provide an experimental analysis of eighty-six scheduling heuristics with scientific workloads of memory and CPU-intensive jobs. The proposed techniques outperform classical solutions in terms of quality of service, energy savings, and completion time by 21.73–43.44%, 44.06–92.11%, and 16.38–24.17%, respectively, leading to a cost-efficient resource allocation for cloud infrastructures.

## I. Introduction

Nowadays, data centers are growing exponentially due to cloud services’ popularization [1]. Cloud Service Providers (CSPs) use this kind of infrastructure to offer different tools and resources. They are mostly grouped into several types of services: Software as a Service (SaaS), Platform as a Service (PaaS), Infrastructure as a Service (IaaS), Storage as a Service (STaaS), Communications as a Service (CaaS), Network as a Service (NaaS), Monitoring as a Service (MaaS), a rapidly grooving Serverless computing, etc. [2]. Virtual Machines (VMs) and Containers (CTs) are the backbones of the virtualized services provided on-demand.

The efficient use of the data center infrastructures is fundamental for users and CSPs. For example, the low utilization of the servers is a critical factor for energy consumption inefficiency. Traditionally, researchers have focused on CPU utilization, where the VM placement problem is usually solved by NP-hard bin-packing. Few studies consider additional factors, such as reducing power consumption and avoiding SLA violations due to resource contention.

From a business perspective, reducing energy consumption leads to a significant reduction of environmental concerns and costs, hence reducing the final price for the user and increasing earns for CSPs [3].

The optimization of energy consumption is a challenge despite the diversity of existing energy management strategies. The energy efficiency of clouds has become a crucial research issue [2], mainly after introducing the green computing paradigm. An underlying technology involved in resource utilization and energy consumption of data centers is virtualization.

Resource virtualization refers to the abstraction of the hardware in a computer. Efficient resource usage, portability, scalability, and fast deployment are advantages of virtualization in the cloud infrastructure. The Virtual Machine Monitor (VMM), also called the hypervisor, is the main component of the hardware virtualization systems. This software allows the simultaneous execution of multiple guest VMs on a single server [4]. Some responsibilities of the VMM are strengthening the isolation between VMs and the management of the hardware resource.

The Operating System-level (OS-level) virtualization can be used either alternatively or in addition to hardware virtualization. Virtual CTs can be created on each OS [5,6]. Nowadays, the containerization technique is a buzzword for the cloud industry, especially in data centers [7]. The CTs are beneficial for CSPs since they can be more densely packed than VMs.

Each CT provides an isolated space for the user by encapsulating a group of processes separated from others in the system. The CTs can share the host kernel, libraries, and binaries, making them adequate technology to be used in scientific workflow platforms [8]. Examples of container implementations include Linux Containers (LXD), Docker, Kubernetes, OpenVZ, Singularity, etc.

In general, efficient job scheduling and load balancing over computational nodes remain challenging in massive, dynamic, elastic, diverse, and heterogeneous computational environments such as clouds.

The introduction of CTs technology brought several advantages to the cloud domain, but relevant topics in its field must be improved. Scheduling, load balancing, security, energy consumption, among others, are current topics of high importance to make cloud environments more efficient and accessible for the users.

Several researchers address the problem of energy consumption and/or makespan in data centers. The speed scaling method is highly used to aboard problems where the main objective is to save the energy subjected to the execution time constraint [8–11].

Unfortunately, these strategies affect the Quality of Service (QoS), which can be expressed using priorities, and Service Level Agreements (SLA), among others. Moreover, priority-based job scheduling introduces additional challenges because jobs with higher priorities must be served before those with lower priorities. Furthermore, priorities might influence the resource assignment, e.g., high-priority jobs might receive higher computing power than lower-priority jobs.

Several contention-aware resource allocation strategies to reduce energy consumption and increase performance are proposed [12–16]. Resource contention emphasizes avoiding co-hosted applications that contend for shared resources. These works study the power consumption in environments with bare metal and VMs infrastructure. However, environments with CTs can increase the number of applications competing for shared resources, increasing energy consumption.

This paper focuses on the online jobs’ allocation that contends for resources in a container-based cloud environment. Our contribution is multifold:

- We present job allocation as the multilevel dynamic bin-packing problem that provides a lightweight runtime solution that minimizes contention and energy consumption while maximizing utilization.
- The energy model considers types of applications and their execution on shared resources generalized by the job concentration paradigm.
- Two and three-level hybrid heuristic algorithms with container selection, capacity distribution, and contention-aware allocation are designed to consolidate resources to maximize utilization and reduce resource contention.
- Extensive experiments with eighty-six scheduling heuristics considering scientific workloads with memory and CPU-intensive jobs demonstrate that proposed techniques outperform classical solutions in terms of energy savings and completion time.

The proposed heuristics use different amounts of information in the allocation process. They improve the performance of well-known heuristics and provide a good compromise between QoS, makespan, and energy. We demonstrate that they are suitable for containers in cloud environments as more efficient and valuable solutions.

The rest of the paper is structured as follows. Section II discusses related work. Section III defines the infrastructure, scheduling, and energy model for allocating jobs into a container-based cloud. Section IV describes the processing speed models of the jobs. Section V discusses job allocation strategies. Section VI introduces the experimental setup. Section VII presents the experimental analysis. Finally, we conclude and discuss future work in Section VIII.

## II. Related work

The interest and usage of container-based technologies in cloud environments have been growing significantly. Several works pointed out that CTs are the future of clouds [17].

Several container technologies were developed, where each of them provides different deployment, management, orchestration, and communication. In this section, we review some of these container-based technologies. Then, we present the latest advances in the area of our interest.

An LXD container [18] is an OS-level virtualization technique that runs multiple isolated Linux systems on a single Linux control host. The LXD resides directly on top of the host kernel. Hence, it does not need any extra abstraction layer or another guest OS. A namespace provides an interface for the Linux kernel features and isolations of resources for each CT. The control group manages the allocation of resources (metering and limiting).

Docker [19] is an open-source container project that simplifies the deployment of services with the methodology of one process per container. The Docker Container Engine, an additional abstraction layer, runs a single application in each virtualized instance. This methodology of execution attaches the CT lifetime with the finish time of the application. Docker daemon constructs a writable layer at the top of the CT read-only image to execute the processes.

Kubernetes [20] is an open-source system for managing the lifecycle of heterogeneous containerized applications (deployment, scaling, orchestration, etc.). The smallest deployable computing unit (POD), created and managed by Kubernetes, encapsulates a set of containers tightly coupled with some shared resources. It groups containers with shared storage/network resources for easy management of logical units of an application. A POD can be replicated along with several machines for scalability and fault tolerance purposes. Still, two services that listen on the same port cannot be deployed inside a POD.

OpenVZ [21] is a container-based virtualization technology to manage multiple secure and isolated Linux containers on a single physical server. Virtual Private Servers (VPSs) and CTs are basic units that share hardware and run on the same OS kernel as the host system. Kernel namespaces allow each CT to have an independent set of resources.

Singularity [22] is an open-source scientific container solution supporting an application in existing and traditional High-Performance Computing (HPC) resources. It offers computing mobility, reproducibility, user freedom, and integration with any scientific computational workflow. A Singularity CT image encapsulates the OS-system environment and all application dependencies necessary to run a defined workflow.

Table 1 shows the relevant characteristics of the related works. It highlights a research gap in the domain of CTs and resource contention. Our approach focuses on the completion time and energy consumption of allocation strategies in container-based cloud environments under resource contention.

**Table 1:** Characteristics of related works.

Ref.	Objectives	Bare metal	VMs	CTs	Contention	Evaluation
[23]	Energy	-	●	●	-	Simulation
[24]	Energy	-	●	●	-	Simulation
[25]	Energy, QoS	-	-	●	-	Real
[26]	Energy, QoS	-	-	●	-	Real
[27]	Energy, QoS	-	●	●	-	Simulation
[28]	Energy, SLA/QoS	-	●	●	-	Simulation
[29]	Energy, performance	-	●	●		Simulation
[30]	Response time	-	-	●	-	Real
[31]	Energy	-	-	●	-	Simulation
[32]	Deadline, cost	-	-	●	-	Simulation
[33]	Cost, QoS	-	●	●	-	Simulation
[12]	Energy	●	●	-	●	Simulation
[14]	Utilization	-	-	-	●	Simulation
[15]	Energy	●	-	-	●	Real
[16]	Accuracy	-	●	-	●	Simulation
[34]	Energy, Utilization	-	●	-	●	Simulation
[35]	Energy, Utilization	-	-	-	●	Real

Xu et al. [23] discuss the brownout model to reduce data center energy consumption. This approach can reduce energy consumption by selectively and dynamically deactivating optional application components. The experimental evaluation considers two types of hosts and four types of VMs.

Xu and Buyya [24] increase the functionality of the brownout to reduce data center energy consumption. They proposed a brownout-based approximate Markov Decision Process approach to improve tradeoffs between energy-saving and user discounts.

Xu et al. [25] propose several scheduling algorithms for managing microservices and CTs to reduce power consumption with QoS constraints. They achieve better performance in both objectives than the baseline algorithms.

Xu and Buyya [26] develop BrownoutCon to deal with overloads and reduce power consumption on cloud systems. It is integrated with Docker Swarm [36] to demonstrate its efficiency in managing containers under several policies.

Gholipoura et al. [27] propose a multi-criteria decision-making method for cloud environments with VMs and CTs. The consolidation defines the migration policy based on the virtual resource, CTs, and VMs.

Piraghaj et al. [28] present a set of allocation policies for energy saving on the Containers as a Service (CaaS) cloud paradigm. The authors propose an architecture and several algorithms to minimize energy consumption while maintaining the required SLA and QoS.

Khan et al. [29] study consolidation algorithms for effective migration of VMs, CTs, and applications to save energy without negatively impacting service performance. The results show the impact between the migration of applications and the migration of VMs, the resource consolidation considering various workloads, resources, and datacenter set-ups.

The cloud-based solutions community has also generated Internet of Things (IoT) research using VMs and CTs. The IoT workloads contain workflows with different types of jobs: CPU Intensive (*CI*), Memory Intensive (*MI*), and Bandwidth Intensive (*BI*) [33].

Celesti et al. [30] study the overhead costs of CT virtualization on an IoT device with a Raspberry Pi and Docker engine. The authors highlight the overhead introduced by container virtualization in a real scenario.

Dambreville et al. [31] discuss energy consumption reduction by introducing a prediction process in the scheduling task. The authors modify the available servers to fit the prediction and schedule all of the jobs on the available servers.

Cui and Xiaoqing [32] propose a scheduling solution in cloud computing for workflow tasks based on genetic algorithms. The fitness function defined the weighted sum of the user-defined deadline and the total cost of the workflow application execution. A top-down leveling is defined as the longest path from the task to the leaf task in the workflow for each task. This level is used as a task priority. According to a descending order of these priorities, the tasks are scheduled for the available hosts.

Tchernykh et al. [33] analyze several solutions for a digital twin workflow allocation in the virtual resource in a cloud infrastructure. The goal is to minimize the rent cost and satisfy the computational resources demand; the workloads include *CI*, *MI*, and *BI* jobs in order to model applications with different requirements. The authors propose allocation algorithms based on heuristics, metaheuristics, and mixed-integer programming to find low-cost solutions.

Several approaches focus on resource contention issues, where co-hosted applications contend for shared resources increasing energy consumption and reducing performance.

Armenta-Cano et al. [12,13] propose a resource allocation model for online energy-aware scheduling with job concentration. The authors characterize the energy consumption of applications and their combinations. It is used for heterogeneous job allocation to avoid resource contention.

Sheikhalishahi et al. [14] propose a multilevel resource contention-aware scheduling for energy-efficient in distributed systems. The authors define a resource contention metric for high-performance computing workloads. The approach models the interaction between system and scheduler information concerning jobs and resources.

Muraña et al. [15] study the power consumption for scientific computing applications in multicore systems. The authors evaluate the power consumption of applications in single and combined executions on Intel and AMD servers. The results indicate a strong dependency between the type of applications and power consumption.

Van Beek et al. [16] develop a CPU-contention predictor for Business-Critical Workloads (BCW) in data centers. It estimates performance degradation for VMs, hence, the risks of SLA violations.

Lovász et al. [34] present an energy-performance aware model for VMs allocation in heterogeneous servers as a variant of the multidimensional vector packing problem. The authors propose a prediction model to estimate performance degradation when different services are consolidated.

Blagodurov et al. [35] propose scheduling strategies to mitigate different resource contention sources on multicore processors. The authors define a classification scheme for contention in a cache space, memory controller, memory bus, and prefetching hardware.

In general, the different types of containers allow running a higher number and variety of applications in a single resource. Moreover, the energy consumption of the system is increased if the applications contend for resources. The following section defines the model to characterize job allocation in a container cloud environment aware of resource contention.

## III. Model

In this section, we formulate the infrastructure, job, and energy consumption models, and define the optimization criteria of the job allocation problem. The main notations are summarized in Table A1 of Appendix.

### A. Scheduling model

The IaaS environment is represented by a set of *M* servers (processors), *P* = {*p*_1_, *p*_2_,…,*p*_*M*_}. Each server *p*_*k*_, for all *k* = 1,…,*M*, has a maximum processing capacity *Q*_*k*_ expressed in Millions of Instructions Per Second (MIPS), and runs a set of *m*_*k*_ containers, $C_{k}=\{c_{1}^{k},c_{2}^{k},\ldots ,c_{m_{k}}^{k}\}$. Each container $c_{i}^{k}$, for all *i* = 1,…,*m*_*k*_, has a processing capacity (CPU quota) $q_{i}^{k}$, expressed in MIPS, such that $\sum_{i=1}^{m_{k}}q_{i}^{k}\leq Q_{k}$.

The total number of containers running in the infrastructure is denoted by $m=\sum_{k=1}^{M}m_{k}.\;J(c_{i}^{k})$ defines the subset of jobs running in the container $c_{i}^{k}$ and $\alpha (c_{i}^{k})$ is the number of SLA violations.

We consider a set of *n* independent jobs, *J* = {*j*_1_, *j*_2_,…,*j*_*n*_} that must be scheduled on the set of containers $C={⋃}_{k=1}^{M}C_{k}$. Each job *j*_*j*_ is described by a tuple (*r*_*j*_, *s*_*j*_, *w*_*j*_, *ρ*_*j*_, *o*_*j*_) that consists of its release time *r*_*j*_≥0, the minimum required processing speed *s*_*j*_ in MIPS, the total amount of work *w*_*j*_ in millions of instructions, the job type *ρ*_*j*_ (*CI* and *MI*), and the priority expressed by an integer value *o*_*j*_.

*r*_*j*_ is not available before the job is submitted, and *ρ*_*j*_ is only used for the system to compute the energy consumption. At any given time *t*, a processing speed $s_{j}^{\prime }(t)$ might be assigned to job *j*_*j*_, when $s_{j}^{\prime }(t)<s_{j}$ then the resource incurs in SLA violations $\alpha (c_{i}^{k})=\alpha (c_{i}^{k})+1$ for *j*_*j*_ in *c*_*i*_.

Additionally, *w*_*j*_ can be used as an estimator of the finish time of *j*_*j*_, concerning $s_{j}^{\prime }(t)$. Several techniques can be used to estimate an accuracy value of *w*_*j*_.

In this paper, we limit its use to identify CTs with a major amount of pendant processing. The idea is to study dynamic performance degradation and energy consumption increase due to shared resource contention and present consolidation heuristics based on contention-aware resource provisioning.

We consider this problem as a special case of multilevel dynamic bin-packing (online and non-clairvoyant). Bins represent CTs, and the jobs define the contribution to CT utilization. An additional element to the traditional dynamic bin-packing focuses on the contention-aware distribution of available processing capacity.

The processing speed of a job can be changed during its execution. It can be increased and reduced but should not be lower than the minimum required processing speed *s*_*j*_ to satisfy SLA. When a job finishes its execution, the available processing capacity of the container is distributed between running jobs according to the strategies described in Section IV.

*C*_*max*_ is the maximum finishing time (completion time or makespan) of all jobs.

Let *f*_*i*_ be the finishing time of *j*_*j*_ job. *C*_*max*_ is defined as:

$$C_{\max}=\max(f_{j})\forall j\in J$$ (1)

The total number of *SLA* violations is calculated as follows:

$$SLAv=\sum_{k=1}^{M}\sum_{i=1}^{m_{k}}\alpha (c_{i}^{k})$$ (2)

In this paper, we consider three conflicting objectives for optimization: *SLAv*, *E*, and *C*_*max*_:

$$\mathrm{minimize}(SLAv,E,C_{\max})$$ (3)

The measure of the energy consumption *E* depends on the model. Traditional energy models do not take into account the types of applications and heterogeneity of workloads.

The next section describes an energy model that considers system performance degradation due to jobs contend for resources [12].

### B. Energy model

Let us describe the energy consumption model used in our experimental evaluation. The power consumption of the system is defined by

$$E=\int_{t=1}^{C_{max}}E^{op}(t)dt$$ (4)

where *E*^*op*^(*t*) is the energy consumption of the infrastructure at time *t* calculated as a sum of the energy consumption $e_{k}^{proc}(t)$ of all individual processors *k* at time *t*:

$$E^{op}(t)=\sum_{k=1}^{M}e_{k}^{proc}(t)$$ (5)

Two types of consumption are distinguished in the processor: static and dynamic. The static one is the power consumed by the component without performing useful work due to the leakage current, also known as idle power or base power *e*^*idle*^. The dynamic energy consumption is produced when an application utilizes components during its execution *e*^*used*^.

The energy consumption of the processor *k* at time *t*, $e_{k}^{proc}(t)$ of Eq (5), is computed as:

$$e_{k}^{proc}(t)=o(t)\left(e_{k}^{idle}+e_{k}^{used}(t)\right)$$ (6)

where *o*(*t*) = 1, if the processor is on at time *t*, and *o*(*t*) = 0, otherwise, and the constant value $e_{k}^{idle}$ does not depend on the time *t*.

The energy consumption of the processor *k* at time *t* depends on its utilization level and concentration of different types of jobs:

$$e_{k}^{used}(t)=(e_{k}^{max}-e_{k}^{idle})*F_{k}(t)*g_{k}\left(\varphi_{CI}^{k}(t)\right)$$ (7)

where $e_{k}^{max}$ defines a constant for the maximum energy consumption of the processor at full capacity, 0≤*F*_*k*_(*t*)≤1 is the portion of the power consumed by different types of jobs, and *g*_*k*_ is a fraction of the total energy consumption introduced by job type combinations.

Known energy models are based on the observation that CPU utilization is highly correlated with overall energy consumption. Linear and nonlinear models are frequently used. In this work, we follow a nonlinear hybrid energy consumption model proposed in [12,13] as a function of CPU utilization and concentration of jobs of different types.

We calculate *F*_*k*_(*t*) as a sum of the portion of the power consumed by each job as follows:

$$F_{k}(t)=\sum_{\forall_{d}}f_{d}^{k}\left(U_{d}^{k}(t)\right),0\leq F_{k}(t)\leq 1,d\in \{CI,MI\}$$ (8)

where $f_{d}^{k}$ defines the fraction of energy consumption of a given job type *d* when its processor utilization is $U_{d}^{k}(t)$ at time *t*.

**Fig 1:**
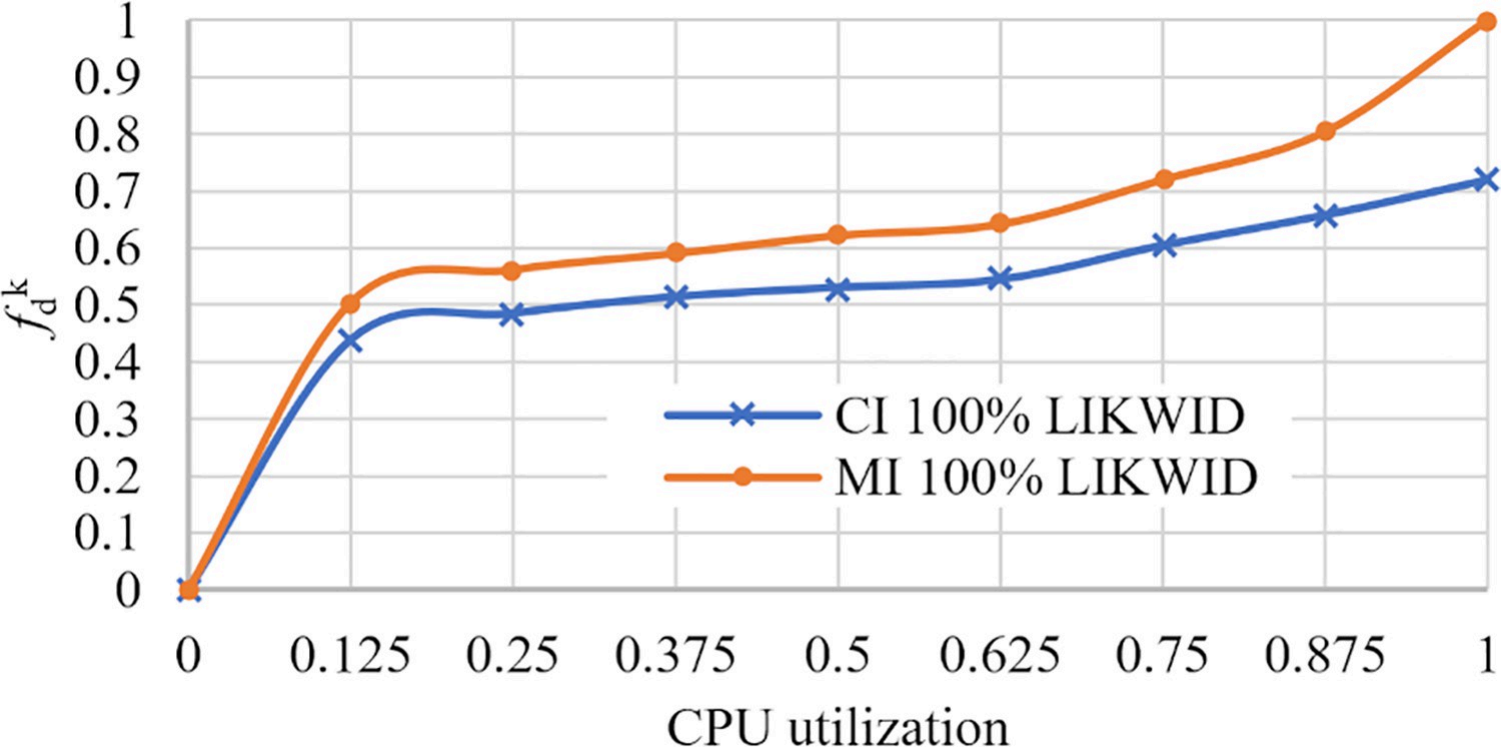
Energy profiles $f_{d}^{k}$ of CI and MI applications.

Fig 1 shows $f_{d}^{k}$ of *CI* and *MI* applications versus CPU utilization. We see that each job type contributes differently to the total energy consumption with the same CPU utilization.

Results are obtained by the well-known performance tool suite LIKWID for the GNU/Linux operating system and SysBench benchmark.

The server used in the experiments is an Express x3650 M4, with two Xeon Ivy Bridge processors E5-2650v2 95W, default clock speed of 2.6 GHz. Each processor has eight cores and two threads per core (16 with hyperthreading), level 1 memory of 32 kB, level 2 of 256 kB, level 3 of 20 MB. Two Non-Uniform Memory Access (NUMA) domains of 32 GB each, with a total memory of 64 GB are used. The server OS is a CentOS Linux release 7.1.1503.

The combination of applications is considered in the following manner. Let us assume two types of jobs. The total CPU utilization is calculated as:

$$U_{T}^{k}(t)=U_{CI}^{k}(t)+U_{MI}^{k}(t)$$ (9)

where $U_{CI}^{k}(t)$ and $U_{MI}^{k}(t)$ are the utilization of all jobs of type *CI* and *MI*, respectively, executed on the processor at time *t*. They are calculated as:

$$U_{CI}^{k}(t)=\frac{\sum_{i=1}^{m_{k}}\sum_{\forall_{j}\in J(c_{i}^{k})}s_{j}^{\prime }(t)}{Q_{k}},\{\rho_{j}=CI\}$$ (10)

$$U_{MI}^{k}(t)=\frac{\sum_{i=1}^{m_{k}}\sum_{\forall_{j}\in J(c_{i}^{k})}s_{j}^{\prime }(t)}{Q_{k}},\{\rho_{j}=MI\}$$ (11)

where $s_{j}^{\prime }(t)$ is a processing speed assigned to job *j*_*j*_ at time *t*.

$g_{k}\left(\varphi_{CI}^{k}(t)\right)$ is a fraction of the total energy consumption introduced by the job types’ combination.

$\varphi_{CI}^{k}(t)$ is the concentration (proportion) of the job *CI* (quantity of job utilization in a processor utilization) at time *t*. It is a measure of the total fraction of CPU capacity consumption of all jobs of a given type in the total capacity.

$$\varphi_{CI}^{k}(t)=\frac{U_{CI}^{k}(t)}{U_{CI}^{k}(t)+U_{MI}^{k}(t)}$$ (12)

$$\varphi_{MI}^{k}(t)=1-\varphi_{CI}^{k}(t)$$ (13)

$g_{k}\left(\varphi_{CI}^{k}(t)\right)=1$ when jobs just with one type are running on the processor.

**Fig 2:**
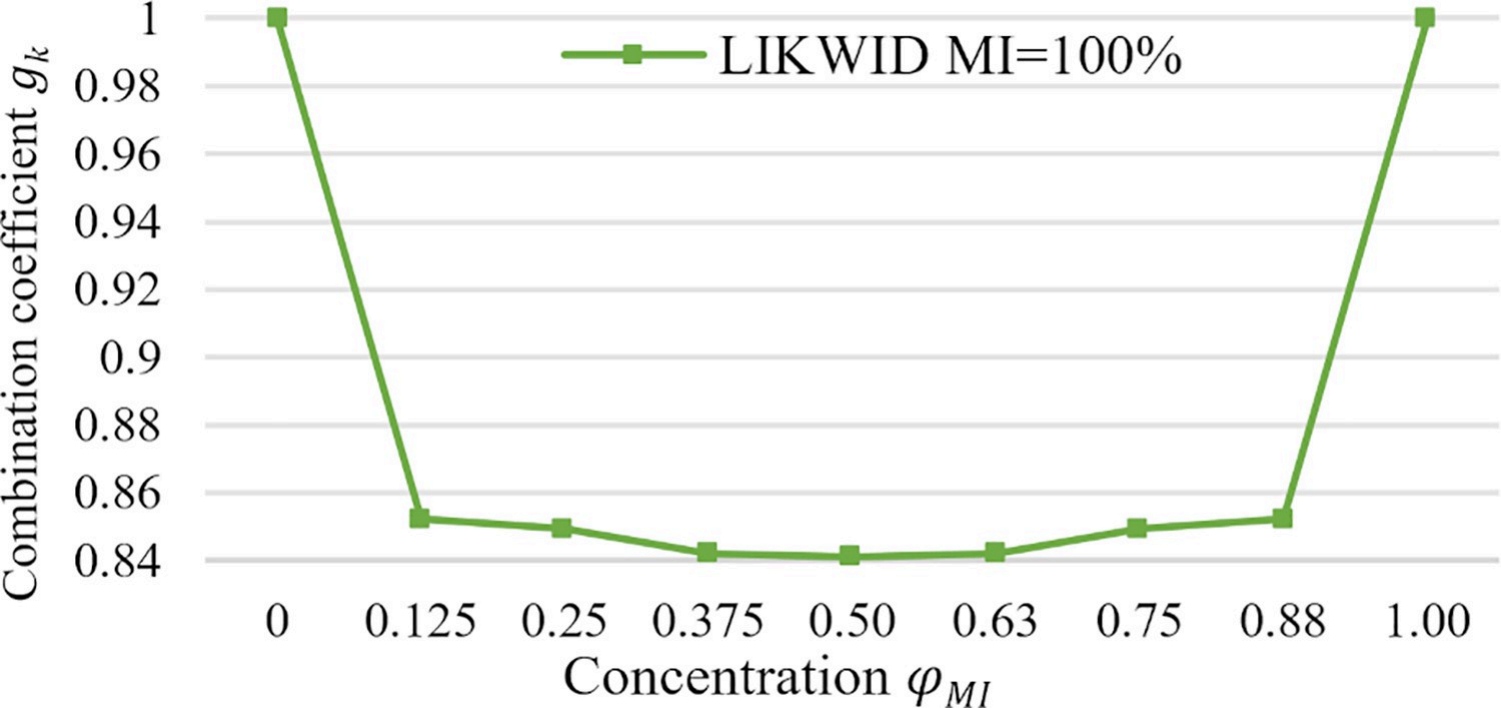
Fraction of energy consumption of two job types.

Fig 2 shows $g_{k}\left(\varphi_{MI}^{k}(t)\right)$ versus $\varphi_{MI}^{k}(t)$ for two job types. We see that *g*_*k*_ = 1 when *MI* jobs are not executed, hence $\varphi_{MI}^{k}(t)=0$ and $\varphi_{CI}^{k}(t)=1$, or only *MI* jobs are executed, hence $\varphi_{MI}^{k}(t)=1$ and $\varphi_{CI}^{k}(t)=1$.

According to Fig 2, we see a reduction in energy consumption when jobs of both types are allocated to the processor.

The coefficient with the lowest energy consumption for *CI* and *MI* concentration is between 0.125 and 0.88. For instance, the concentration 0.125 is obtained for one *MI* and seven *CI* applications per eight-core processor. The concentration 0.88 is obtained for seven *MI* and one *CI* applications. If both concentrations are about 0.5, *g*_*k*_ = 0.84.

The concentration $\varphi_{CI}^{k}(t)=0$ implies that all applications in the processor are *MI* type, $\varphi_{MI}^{k}(t)=1$, and vice versa. In both cases, there is no reduction in energy consumption because $g_{k}\left(\varphi_{CI}^{k}(t)\right)=1$.

A Lagrange interpolating polynomial represents the energy profile of each job type [15]. We use the obtained equations to estimate the energy consumption of jobs in the energy model.

## IV. Processing capacity model

In this section, we focus on three models of the processing capacity distribution between jobs during allocation and execution: required capacity, full proportional capacity, and full priority capacity.

## A. Required capacity

In the Required Capacity model (*RC*), the assigned job speed $s_{j}^{\prime }(t)$ cannot be higher than the minimum required processing speed *s*_*j*_ to satisfy SLA.

The main idea of the RC model is to limit the utilization of CTs to avoid that jobs conflict for shared physical resources. We limit the utilization of the CTs to avoid the saturation of the resource due to several CTs can be hosted in it.

The threshold of the job allocation and available capacity redistribution is the minimum processing speed of each job. Formally,

$$s_{j}^{\prime }(t)=q_{i}^{k}*v_{j},\;\mathrm{where}\{\begin{array}{l} v_{j}=1,\;\mathrm{if}\;\sum_{j\in J(c_{i}^{k})}s_{j}\leq q_{i}^{k},\\ v_{j}=\frac{s_{j}}{\sum_{j\in J(c_{i}^{k})}s_{j}},\;\mathrm{other}\;\mathrm{cases}. \end{array}$$ (14)

**Fig 3:**
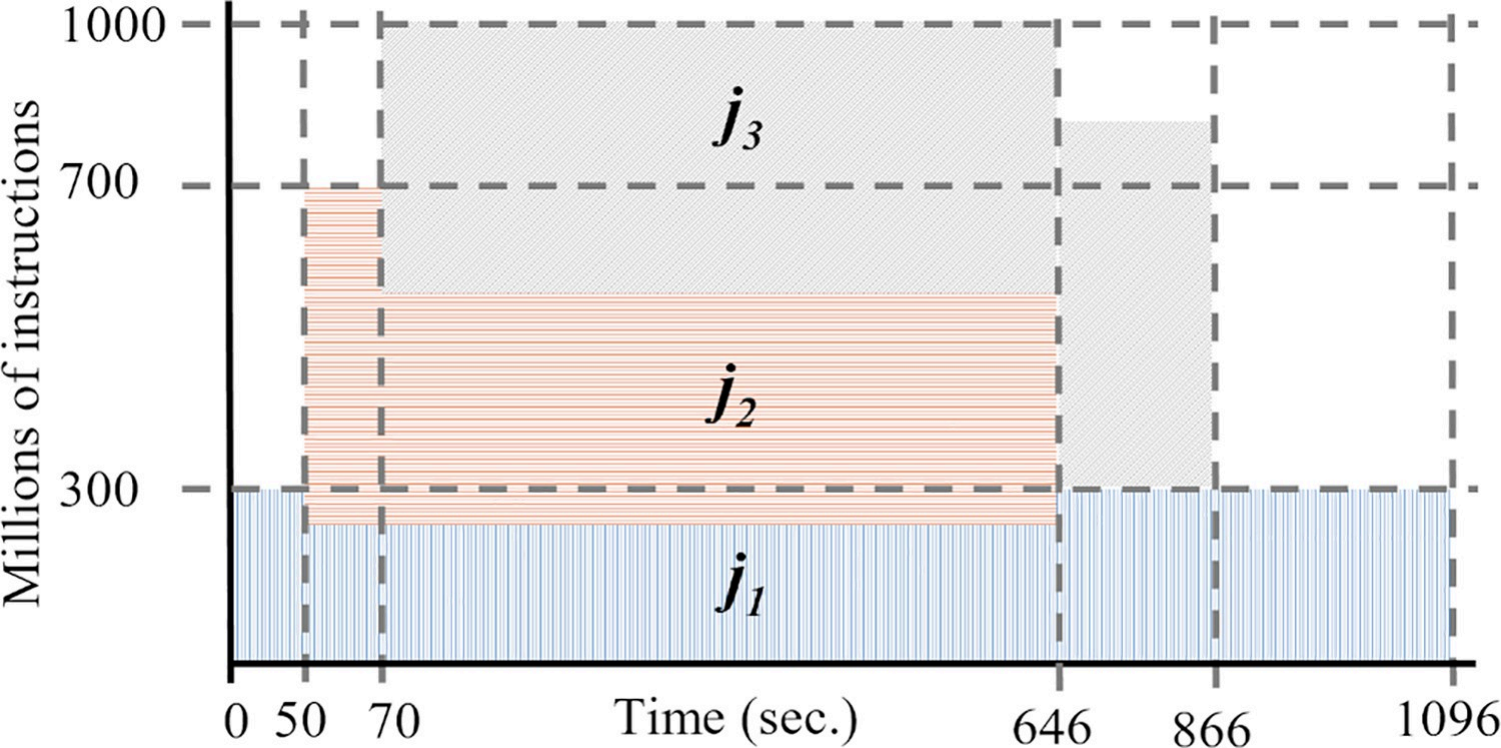
Example of container capacity distribution with the required capacity model.

Fig 3 illustrates an example of the job allocation with the *RC* model. We consider three jobs with the following specifications: *j*_1_ = (0, 300, 3*10^14^, 1), *j*_2_ = (50, 400, 2*10^14^, 3), *j*_3_ = (70, 500, 3.5*10^14^, 2), and container $c_{j}^{k}$ with capacity $q_{i}^{k}=1,000$ MIPS.

Initially, the scheduler defines the processing speed of *j*_1_ to *s*′_1_(0) = 300 because it is available at time 0. When *j*_2_ arrives, at time *t* = 50, the scheduler assigns the required processing speed *s*′_2_(50) = 400. At time *t* = 70, job *j*_3_ cannot be allocated in the container because $c_{i}^{k}$ does not have enough available resources to fulfill *s*′_3_(70) = 500. So, the processing capacity $q_{i}^{k}$ is distributed using Eq (14), violating SLA.

Job *j*_2_ ends its execution at the time *f*_2_ = 646, and the scheduler updates the speed of jobs in the container, *s*′_1_(646) = 300 and *s*′_3_(646) = 500. The finish time of jobs are *f*_3_ = 866, and *f*_1_ = 1,096.

The two additional models keep resource utilization in full. The only difference is the distribution of the exceeding capacity, additional capacity after satisfying the basic requirement of jobs running in the CT.

### B. Full proportional capacity

In the Full Proportional capacity model (*Prop*), unallocated available capacity in the container is distributed proportional among jobs. Formally, we assume that *j*_*i*_ is assigned to the container $c_{j}^{k}$.

$$s_{j}^{\prime }(t)=q_{i}^{k}*v_{j},\;\mathrm{where}\;v_{j}=\frac{s_{j}}{\sum_{b\in J(c_{i}^{k})}s_{b}}$$ (15)

**Fig 4:**
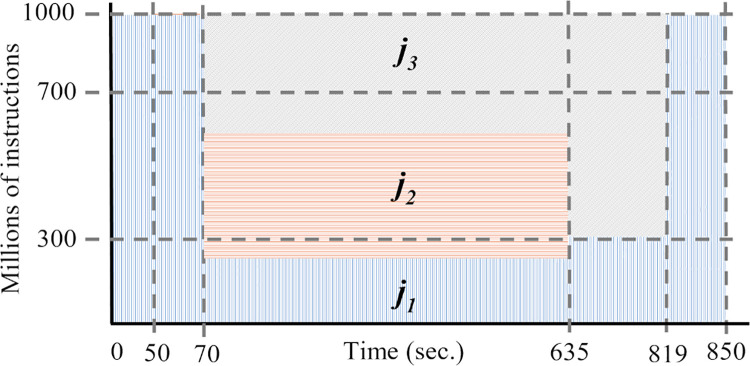
Example of container capacity distribution with a full proportional model.

Fig 4 shows an example of allocation jobs with *Prop* model. Similar to the previous example, we consider the three jobs *j*_1_, *j*_2_, and *j*_3_ and one container $c_{i}^{k}$.

Job *j*_1_ is available at time 0, with the *Prop* model, *j*_1_ can use the full capacity of the container *s*′_1_(0) = 1,000. Job *j*_2_ arrives at time *t* = 50, the capacity of $c_{i}^{k}$ is divided between both jobs according to Eq (15), so the scheduler defines the jobs processing speed with *s*′_1_(50) = 429 and *s*′_2_(50) = 571. During the execution of job *j*_3_, from *t* = 70 to *t* = 635, the speed values for the three jobs in the container are 250, 334, and 416, respectively. Job *j*_2_ ends its execution at a time *f*_2_ = 635 and the speed of the jobs in the container are updated to *s*′_1_(635) = 375, and *s*′_3_(635) = 625. The finish times of jobs *j*_3_, and *j*_1_ are *f*_3_ = 819, and *f*_1_ = 850.

### C. Full priority capacity

In the Full Priority capacity model (*Prio*), unallocated or available capacity in the container is distributed between jobs based on their priorities. Formally, it assumes that *j*_*j*_ is assigned to the *i* container,

$$s_{j}^{\prime }(t)=factor*o_{j}*q_{i}^{k},\forall J_{j}\in J(c_{i}^{k})\cup J_{j}$$ (16)

where

$${factor}_{c_{i}^{k}}=\frac{1}{\left(o_{j}+\sum_{J_{j}\in J(c_{i}^{k})}o_{j}\right)}$$ (17)

**Fig 5:**
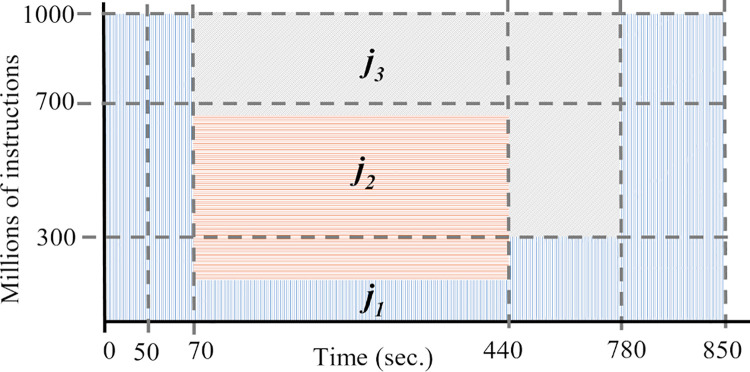
Example of container capacity distribution with the full priority model.

Fig 5 presents an example of allocation jobs with the *Prio* model. Similar to the two previous examples, we consider three jobs *j*_1_, *j*_2_, *j*_3_, and one container $c_{i}^{k}$.

The scheduler assigns the total CPU capacity to job *j*_1_ at time 0, *s*′_1_(0) = 1,000. Job *j*_2_ arrives at time *t* = 50, then the capacity of $c_{i}^{k}$ is distributed between both jobs according to Eq (16), the speed of both jobs is set up to *s*′_1_(50) = 250 and *s*′_2_(50) = 750. When job *j*_3_ arrives, the scheduler reassigns the CPU capacity and the values of processing change to *s*′_1_(70) = 167, *s*′_2_(70) = 500, and *s*′_3_(70) = 333. Job *j*_2_ ends its execution at the time *f*_2_ = 440 and the CPU capacity is redistributed between jobs *j*_1_ and *j*_3_, in this way *s*′_1_(440) = 333 and *s*′_3_(440) = 667. After 340 seconds, job *j*_3_ finishes its execution and all the CPU capacity is reassigned to *j*_1_. The finish time of both jobs are *f*_3_ = 780 and *f*_1_ = 850. Note that two of the three jobs finish their execution before the previous examples.

## V. Job allocation

This section describes the set of scheduling heuristics for job allocation. They are similar to the well-known dynamic bin-packing problem, a variation of the classical NP-hard optimization problem.

Several heuristics were proposed to affront bin-packing problems producing solutions in a reasonable time frame. Both time and performance are fundamental in online resource allocation. Therefore, we propose heuristic-based scheduling strategies for the job allocation to CTs.

The scheduler performs two main tasks: CT selection and CPU assignation. In the selection stage, the scheduler decides whether the job is placed into one of the available CTs or a new CT should be invoked.

In the CPU assignation stage, the scheduler establishes the capacity inside the CT to execute the job. We use different factors in the system to allocate the jobs in the CT selection stage.

### A. Container selection strategies

As an example, we assume that the scheduler follows the *RC* model. According to this model, the processing speed $s_{j}^{\prime }(t)$ assigned to a job *j*_*j*_ at time *t* must never be higher than the minimum required processing speed *s*_*j*_, i.e., $s_{j}^{\prime }(t)\leq s_{j}$.

The first two baseline strategies are:

- Random (Rand)–the scheduler allocates a job to a random container $c_{i}^{k}\in C,\;1\leq k\leq M,\;1\leq i\leq m_{k}$.
- Round Robin (RR)–the scheduler allocates a job using a Round Robin strategy, distributing to containers in rotation.

These strategies are “blind” because they do not use specific information about job allocations. Other strategies take into account information about the job to be assigned and CTs.

We consider three scenarios in the selection of a CT:

1. At least one container has sufficient capacity to execute the arriving job without SLA violation.
2. At least one container is not running at its full capacity.
3. All containers are using their maximum capacity.

Several policies can be used to face each scenario. A specialized policy per scenario can provide strategies with better results.

Whenever a job arrives, the scheduler uses the first policy to select a CT with enough capacity to execute it. If the job cannot be allocated without SLA violation, the scheduler employs a second policy to choose between CTs with available capacity. Finally, if all containers are running at full capacity, the scheduler uses the third policy to choose a CT.

Table 2 presents the selection policies of CTs that have sufficient capacity to execute the arriving job. Table 3 shows the policies to select one CT among sufficient capacity containers. The preferred candidates are those that are not running at their full capacity.

**Table 2:** Selection policies of containers with sufficient capacity.

Strategy	Description
First Fit (FFit)	Allocates job *j*_*j*_ to the first container capable of executing it, the first CT that satisfies $q_{i}-(\sum_{\forall_{d}\in J(c_{i})}s_{d}+s_{j})\geq 0$ for *c*_*i*_.
Best Fit (BFit)	Allocates job *j*_*j*_ to the container with the smallest utilization left, $Min\left(q_{i}-(\sum_{\forall_{d}\in J(c_{i})}s_{d}+s_{j})\right)$ for *c*_*i*_.
Worst Fit (WFit)	Allocates job *j*_*j*_ to the container with the largest utilization left, $Max\left(q_{i}-(\sum_{\forall_{d}\in J(c_{i})}s_{d}+s_{j})\right)$ for *c*_*i*_.
Minimum Amount of Work (MinAW)	Allocates job *j*_*j*_ to the container with the minimum total amount of pending work for jobs running in the container, $Min(\sum_{\forall_{d}\in J(c_{i})}w_{d}^{\prime })$ where $w_{d}^{\prime }$ defines the amount of work processed of *j*_*d*_ from *r*_*d*_ to *t* in *c*_*i*_.
MaxSLAv	Allocates job *j*_*j*_ to the container with more SLA violations, *Max*(*α*(*c*_*i*_)) for *c*_*i*_.
MinSLAv	Allocates job *j*_*j*_ to the container with less SLA violations, *Min*(*α*(*c*_*i*_)) for *c*_*i*_.
Rand	Allocates job *j*_*j*_ randomly to an active CT that satisfies $q_{i}-(\sum_{\forall_{d}\in J(c_{i})}s_{d}+s_{j})\geq 0$ for *c*_*i*_.

**Table 3:** Selection policies of containers with available capacity.

Strategy	Description
MinTask	Allocates job *j*_*j*_ to the container with the minimum number of assigned jobs, *Min*(*J*(*c*_*i*_)) for *c*_*i*_.
MaxCap	Allocates job *j*_*j*_ to the container with maximum capacity available, $Min(q_{i}-\sum_{\forall_{d}\in J(c_{i})}s_{d})$ for *c*_*i*_.
MinCap	Allocates job *j*_*j*_ to the container with minimum capacity available, $Max(q_{i}-\sum_{\forall_{d}\in J(c_{i})}s_{d})$ for *c*_*i*_.
MinSLAv	Allocates job *j*_*j*_ to the container with less SLA violations at time *t*, *Min*(*α*(*c*_*i*_)) for *c*_*i*_.

Finally, we use two policies for CT selection when all CTs are working with full capacity: MinTasks and MinSLAv. Both strategies are described in Table 3.

### B. Resources allocation strategies

Once a CT is selected, the scheduler assigns the CPU capacity to a job inside the selected CT. If the CT is selected with enough capacity, there are no SLA violations; otherwise, SLA violations can occur.

The capacity assignment is provided by the three models defined in section IV.

1. Required capacity model (*RC*).
2. Proportional capacity model (*Prop*).
3. Priority capacity model (*Prio*).

*RC* strategies combine three policies to allocate jobs to CTs. For instance, FFit–MinTask–MinSLAv strategy selects between CTs with enough capacity using the FFit policy. MinTask policy is used when some CTs have free capacity but not enough to satisfy the capacity requested by the job, and MinSLAv policy selects CT at maximum capacities. The *RC* strategy reassigns capacity when it is overpassed by the requested capacity of the jobs.

As an example, Algorithm 1 presents the FFit–MinTask–MinSLAv strategy.

The worst-case occurs when all CTs are full and active. Then the scheduler searches in the list of active CTs to allocate the job (line 2) with complexity *O*(*k*) for k containers. It cannot assign the job at this stage, so it uses the second strategy (line 8) with the same complexity *O*(*k*). Finally, the strategy runs the procedure in line 10 with *O*(*k*) complexity. The procedure is performed each time a job arrives, so the algorithm has an asymptotical complexity *O*(*nk*).

**Algorithm 1**: **FFit – MinTask – MinSLAv strategy**

**Input:** List of active CTs (*CT*_*A*_) and job *j*_*i*_

**Output:** Index of container to execute job *j*_*i*_

1.*ctIndex* ← -1

2.*ctIndex* ← *FFit* (*CT*_*A*_, *j*_*i*_)

3.        **if** *ctIndex* < 0 **then**

4.            if can create a new]

5.container is true **then**

6.                create a new

7.container *nCT*

8.                add *nCT* to *CT*_*A*_ **and**

9.*ctIndex* ← index of *nCT*

10.                **else**

11.                        ctIndex ← *MinTask*

12.(*CT*_*A*_, *j*_*i*_)

                            **if** ctIndex < 0

    **then**

                                ctIndex ←

    *MinSLAv* (*j*_*i*_)

    reassign CPU speed in

    container ctIndex with *RC*

    **return** *ctIndex*

In *Prop* and *Prio* strategies, the CT selection is a combination of two policies. CT always has full capacity, but the strategy can estimate if an allocation generates SLA violation. For instance, with the FFit–MinTask–Prio strategy, the scheduler selects a CT using FFit strategy as a first policy and MinTask as the second policy. Once a container has been selected, *Prio* model reassigns the processing capacity using Eq (16). Algorithm 2 presents the FFit–MinTask strategy.

Similar to Algorithm 1, the complexity of Algorithm 2 is bounded by *O*(*k*) in lines 2 and 8, so the complexity of the procedure is defined by *O*(*nk*). Note, if we can create an unlimited number of CTs in the infrastructure then both procedures have an asymptotical factor of (*n*^2^) in the worst-case.

**Algorithm 2: FFit – MinTask strategy**

**Input**: List of active CTs (*CT*_*A*_) and job *j*_*i*_

**Output**: Index of container to execute job *j*_*i*_

    *ctIndex* ← -1

    *ctIndex* ← *FFit* (*CT*_*A*_, *j*_*i*_)

        **if** *ctIndex* < 0 **then**

1.            **if** can create a new

2.container is true **then**

3.                create a new

4.container *nCT*

5.                    add *nCT* to *CT*_*A*_

6.and *ctIndex* ← index of *nCT*

7.                **else**

8.            *ctIndex* ← *MinTask*

9.(*j*_*i*_)

10.reassign CPU speed in

    container *ctIndex* with *Prio*

    **return** *ctIndex*

## VI. Experimental setup

All experiments are performed on the standard trace-based simulator CloudSim v3.0.3 [37]. It supports the modeling and simulation of large-scale cloud computing environments. CloudSim includes classes and interfaces such as data centers, single computing nodes, an autonomous platform for modeling clouds, service intermediaries, provisioning policies, allocation strategies, among others.

We extended its functionality with our scheduling algorithms, energy model, dynamic jobs arrival, container deployment, statistical analysis, and workload processing. The simulator represents an excellent tool to develop our experiments [38].

Algorithms are implemented using jdk 1.8.0_221 64-bit. The execution was performed by a computer with Windows 10 Pro OS, an Intel (R) Core (TM) i5-8400HQ CPU 2.8 GHz, 8 GB of memory, and 500 GB of HDD.

### A. Scenario

We use a two-tier topology with *M* processors at the first level and *m* containers at the second level. We perform an experimental analysis with a limited but a representative number of physical resources. In the experimental environment based on CTs on bare metal, two CTs can be executed in one physical resource. Under this scenario, the jobs of both CTs contend for the underlay resources.

The setup defines a basic cloud environment that simplifies the analysis and evaluation of the proposed strategies and maintains the relevant elements of real environments. This configuration can be generalized considering the characteristics and size of resources, CTs capabilities, and workloads. Moreover, the energy model of specific resources and job energy profiles (i.e., *BI*) and their combination (concentration).

*e*^*idle*^ and *e*^*max*^ values are defined according to [12,13]. The collected data from a Power Distributor Unit (PDU) showed that the *e*^*idle*^ of the server is 86 Watts (W) on average. A similar procedure under a fully busy processor was performed to find *e*^*max*^. Table 4 presents the experimental setup.

**Table 4:** Experimental setup.

	Procs	Container
Number of resources	25	50
Cores	1	1
MIPS	1,000	500
Memory	1,000	500
*e*^*idle*^ (W)	86	-
*e*^*max*^ (W)	180	-
Type	-	OS

### B. Workload

The workload is based on HPC traces of parallel workloads [39] and grids from Grid Workload Archive [40]. We use the Standard Workload Format (SWF) with two additional fields to describe the requested work speed and the type of work. Several filters were applied to workloads to eliminate jobs with inconsistent information.

The performance of our strategies was evaluated in a homogeneous resource environment using 30 workloads of 24 hours to obtain valid statistical values. Variability of the arriving time, size, and types in the workload is fundamental to evaluate our strategies’ efficiency under different scenarios properly.

**Fig 6:**
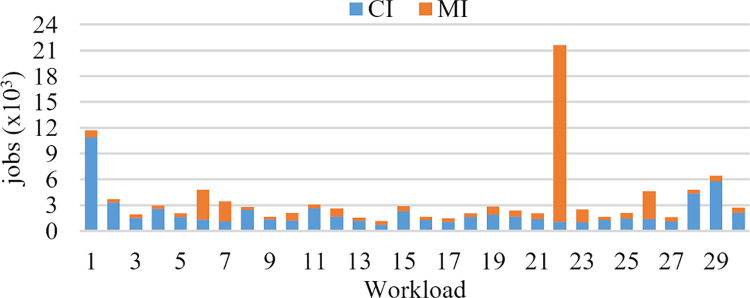
Number and type of jobs per day.

Fig 6 shows the number of jobs of two types during 30 days. We observe that there is no predominance of one type of job in logs. Some weeks have more jobs of type *CI*, and others more jobs of type *MI*. The total number of jobs in the workload is 109,345. Day twenty-two has the biggest workload among all, with more than 20,000 jobs.

*CI* jobs request high computational power in their processing, e.g., calculate prime numbers, sorting, search, graph traversal, etc. In *MI* jobs, the job processing is limited by the speed of memory to feed data to the processor, e.g., work with datasets much larger than the available cache on the system.

**Fig 7:**
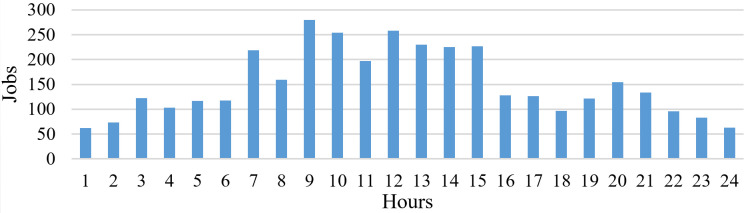
Online distribution of jobs per hour.

Fig 7 shows a histogram with the total number of jobs arriving per hour on an average of 30 days. It can be seen that most jobs arrive between 7 AM and 3 PM.

### C. Analysis methodology

The optimization problem is to minimize three conflict objectives: *SLAv*, *C*_*max*_, and *E*, see Eq (3). In multi-objective optimization, one solution can represent the best solution concerning *C*_*max*_, while another solution could be the best one concerning *E* or *SLAv*. The goal is to obtain a set of compromise solutions that represents a good approximation to the Pareto front.

If a solution cannot be improved in terms of all objective functions, then the outcome is Pareto optimal. Pareto fronts can be compared via visual observation of the solution space and formal approaches.

Set Coverage (*SC*) metric is a formal and statistical approach to calculate the proportion of solutions dominated between two sets [41]. Larger values of *SC* represent a better approximation of the Pareto front. The dominance operator is not symmetric, so it is necessary to compute the dominance of one set over the other and vice versa.

A multi-objective optimization problem can be simplified to a single objective through different methods. The prevalent approach is the aggregation method, which defines weights for each objective to model preferences and performs a single weighted sum. That is, the preferences can explicitly specify the importance of every criterion or relative importance between criteria.

The *degradation* in performance (relative error) indicates the ratio of a metric generated by the algorithms to the best-found solution [42]. The analysis is conducted as follows. First, the degradation in performance of each strategy is computed as follows:

$$\frac{strategy\;criterion\;value}{best\;found\;criterion\;value}-1$$ (18)

Then, the strategies are ranked based on the average of their computed values considering all the scenarios. The best strategy with the lowest average performance degradation has a rank of 1. Low ranks identify strategies that perform reliably well over different scenarios, they represent a good compromise for all test cases.

One disadvantage of the *degradation* approach is to use the mean values to analyze the results. Hence, they can be influenced by a small portion of data with a large deviation. For deeper analysis, we present the strategies’ performance profiles to help with the interpretation of the data.

The Performance Profile (*PP)* defines a non-decreasing, piecewise constant function *δ*(*τ*) that presents the probability that a ratio τ is within a factor of the best ratio. The function *δ*(*τ*) is the cumulative distribution function. Strategies with a large probability for small τ are to be preferred [43].

To choose an adequate strategy, we compare the performance of all strategies by the three analysis methodologies: *degradation*, *PP*, and *SC*.

## VII. Experimental evaluation

In this section, we present results of evaluation of 86 strategies. Their names are combinations of strategies on the first, second, third levels, and capacity models. Table 5 shows 56 strategies for *RC* model. Table 6 shows 14 strategies that can be combined with *Prop* and *Prio* model (28 in total), Random (Rand), and Round Robin (RR).

**Table 5:** Strategies for *RC* model.

No.	Level			No.	Level		
	1	2	3		1	2	3
1	First Fit (FFit)	MinTask	MinTasks	33	MaxSLAv	MinTask	MinTasks
2		MaxCap		34		MaxCap	
3		MinCap		35		MinCap	
4		MinSLAv		36		MinSLAv	
5		MinTask	MinSLAv	37		MinTask	MinSLAv
6		MaxCap		38		MaxCap	
7		MinCap		39		MinCap	
8		MinSLAv		40		MinSLAv	
9	Best Fit (FFit)	MinTask	MinTasks	41	MinSLAv	MinTask	MinTasks
10		MaxCap		42		MaxCap	
11		MinCap		43		MinCap	
12		MinSLAv		44		MinSLAv	
13		MinTask	MinSLAv	45		MinTask	MinSLAv
14		MaxCap		46		MaxCap	
15		MinCap		47		MinCap	
16		MinSLAv		48		MinSLAv	
17	Worst Fit (WFit)	MinTask	MinTasks	49	Rand	MinTask	MinTasks
18		MaxCap		50		MaxCap	
19		MinCap		51		MinCap	
20		MinSLAv		52		MinSLAv	
21		MinTask	MinSLAv	53		MinTask	MinSLAv
22		MaxCap		54		MaxCap	
23		MinCap		55		MinCap	
24		MinSLAv		56		MinSLAv	
25	Minimum Amount of Work (MinAW)	MinTask	MinTasks				
26		MaxCap					
27		MinCap					
28		MinSLAv					
29		MinTask	MinSLAv				
30		MaxCap					
31		MinCap					
32		MinSLAv					

**Table 6:** Strategies for *Prop* and *Prio* models.

No.	Level		model
	1	2	
1	First Fit (FFit)	MinTask	*Prop*
2		MinSLAv	
3	Best Fit (BFit)	MinTask	
4		MinSLAv	
5	Worst Fit (WFit)	MinTask	
6		MinSLAv	
7	Minimum Amount of Work (MinAW)	MinTask	
8		MinSLAv	
9	MaxSLAv	MinTask	
10		MinSLAv	
11	MinSLAv	MinTask	
12		MinSLAv	
13	Rand	MinTask	
14		MinSLAv	
15	First Fit (FFit)	MinTask	*Prio*
16		MinSLAv	
17	Best Fit (BFit)	MinTask	
18		MinSLAv	
19	Worst Fit (WFit)	MinTask	
20		MinSLAv	
21	Minimum Amount of Work (MinAW)	MinTask	
22		MinSLAv	
23	MaxSLAv	MinTask	
24		MinSLAv	
25	MinSLAv	MinTask	
26		MinSLAv	
27	Rand	MinTask	
28		MinSLAv	

The performance of our strategies is evaluated based on the simulation of 30 days of 24 hours each. Runtime of one strategy simulation of the 24 hours workload takes about 1–2 second, on average.

Let us consider the best and worst performance strategies for each objective independently. The results of other strategies were omitted for simplicity and clarity.

**Fig 8:**
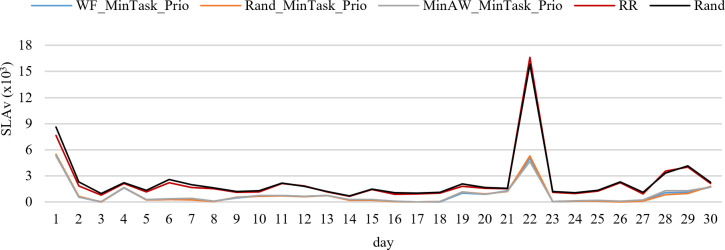
*SLAv* for the best and worst strategies.

Fig 8 presents *SLAv* for the best and worst strategies during 30 days on 24 hours average. WF_MinTask_Prio has the best performance. Its *SLAv* varies from 27 to 5,294 per day. *SLAv* of WF_MinTask_Prop varies from 0 to 7,752. RR and Rand are the worst strategies, their *SLAv*s vary from 636 and 716 to 16,653 and 15,869, respectively.

**Fig 9:**
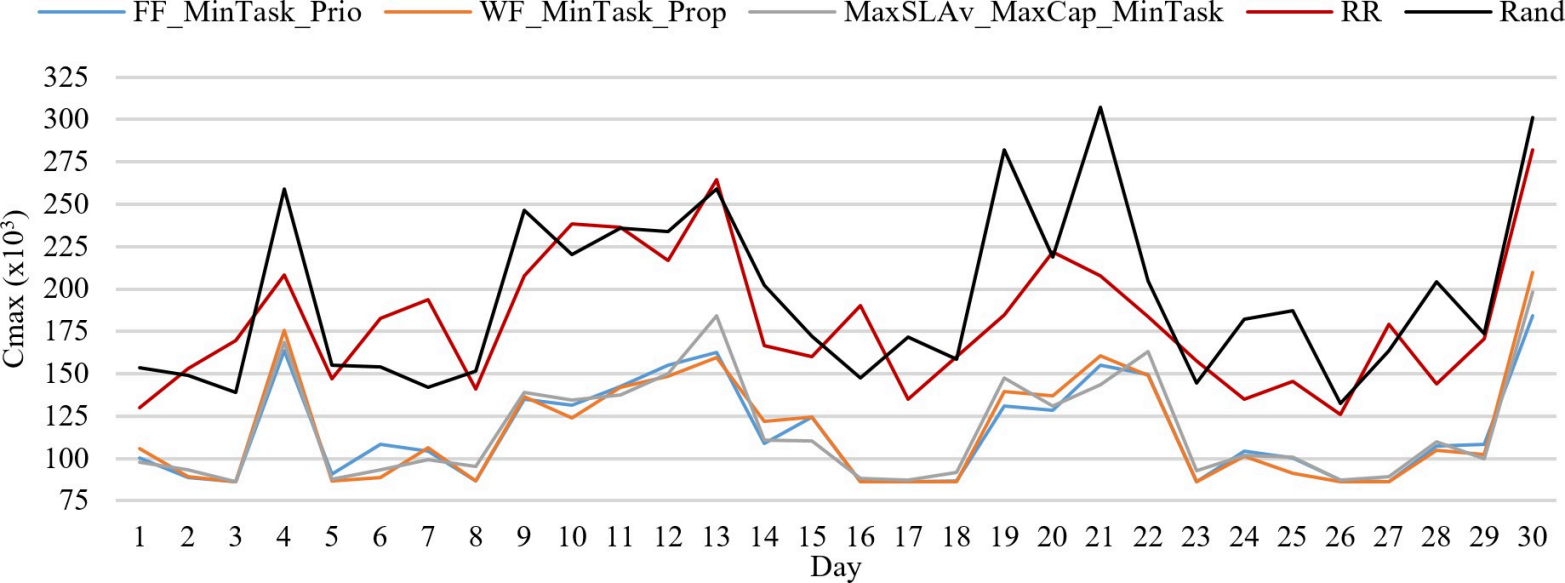
*C*_*max*_ for the best and worst strategies.

Fig 9 shows *C*_*max*_ of the best and worst strategies during 30 days on 24 hours average. Comparing these strategies, we see that the total *C*_*max*_ of all jobs of a day varies between 32.33 and 54.44 hours on average. MaxSLAv_MaxCap_MinTask, FF_MinTask_Prio, and WF_MinTask_Prio are the best strategies showing a similar behavior. RR and Rand are the worst strategies.

**Fig 10:**
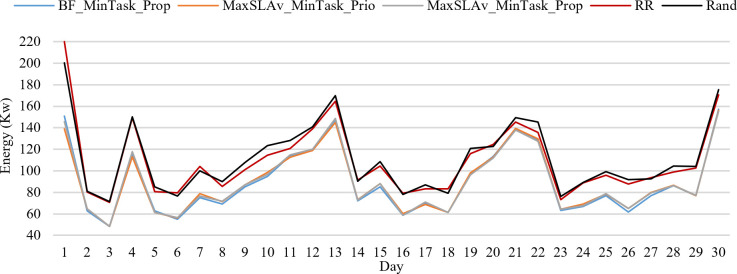
*E* for the best and worst strategies.

Fig 10 presents *E* during 30 days on 24 hours average. BF_MinTask_Prop is the best strategy. It provides allocation of jobs that consume between 48 and 157 Kw per day. The worst two strategies are RR and Rand with energy consumption between 70 and 71 to 200 and 220 Kw per day, respectively.

### A. Degradation in performance analysis

Table 7 presents the average degradation of *SLAv*, *E*, *C*_*max*_, and their means, see Eq (18). The last five columns contain the ranking of *SLAv*, *E*, *C*_*max*_, Rank mean, Rank, their means, and the final ranking. Rank *SLAv* is based on the number of *SLA* violations. Rank *E* depends on the energy degradation. Rank *C*_*max*_ refers to the position to the completion of the total jobs. The most significant values are highlighted in bold and red.

**Table 7:** Average degradation and ranking for the best strategies.

Strategy	*SLAv*	*E*	*C*_*max*_	Mean	Rank *SLAv*	Rank *E*	Rank *C*_*max*_	Rank mean	Rank
WF_MinTask_Prop	0.223	0.047	0.087	0.119	**1**	14	**1**	**1**	**2**
MinAW_MinTask_Prop	0.247	0.045	0.103	0.132	**2**	10	8	**2**	**3**
MinSLAv_MinTask_Prop	0.723	0.040	0.126	0.296	**3**	4	21	4	5
BF_MinTask_Prop	0.781	0.027	0.103	0.304	5	**1**	9	5	**1**
MaxSLAv_MinTask_Prio	11.545	0.037	0.104	3.895	45	**2**	10	45	12
MaxSLAv_MinTask_Prop	0.729	0.038	0.120	0.295	4	**3**	19	3	4
FF_MinTask_Prio	8.152	0.046	0.087	2.762	35	11	**2**	35	9
MaxSLAv_MaxCap_MinTask	11.820	0.053	0.096	3.990	49	24	**3**	47	19
Rand	129.551	0.299	0.837	43.562	86	86	86	86	78
RR	122.365	0.278	0.724	41.122	85	85	85	85	77

The Rank refers to the relative position to all 86 strategies. It considers the mean of *SLAv*, *E* and *C*_*max*_. The best strategies in each criterion are highlighted.

For *SLA* violations, the best strategies are WF_MinTask_Prop, MinAW_MinTask_Prop, and MinSLAv_MinTask_Prop with 22.3%, 24.7%, and 72.3% away from the best solutions found, respectively. RR and Rand are 12,236.5% and 12,955.1% worse.

For energy consumption, the best strategy is BF_MinTask_Prop, with an average *E* degradation 0.027. It allocates the arriving job to the container with the smallest available capacity. If any container has enough space to provide the required speed to the job, the scheduler selects the container with the least number of tasks. MaxSLAv_MinTask_Prio and MaxSLAv_MinTask_Prop follow the best strategy with degradation 0.037 and 0.038, respectively.

For *C*_*max*_ degradation, WF_MinTask_Prop is the strategy with the best performance with a degradation of 0.087. The arriving job is assigned to the container with the biggest available capacity. At the second level of assignation, it selects the container with the least number of tasks. The second and third best strategies with degradations of 0.87 and 0.96 are FF_MinTask_Prio and MaxSLAv_MaxCap_MinTask.

According to the final Rank, strategies with a good compromise between *SLAv*, *E* and *C*_*max*_, are BF_MinTask_Prop, MinAW_MinTask_Prop, and MaxSLAv_MinTask_Prop. The final Rank of RR is 85. It means that at least 84 strategies performed better than RR. Rand and RR provide 129,551% of worse solutions with respect to the best solutions found in the simulation. While, BF_MinTask_Prop is away from the best solutions between 78.1% for *SLA*, 2.7% for *E*, and 10.3% for *C*_*max*_.

### B. Performance profile analysis

**Fig 11:**
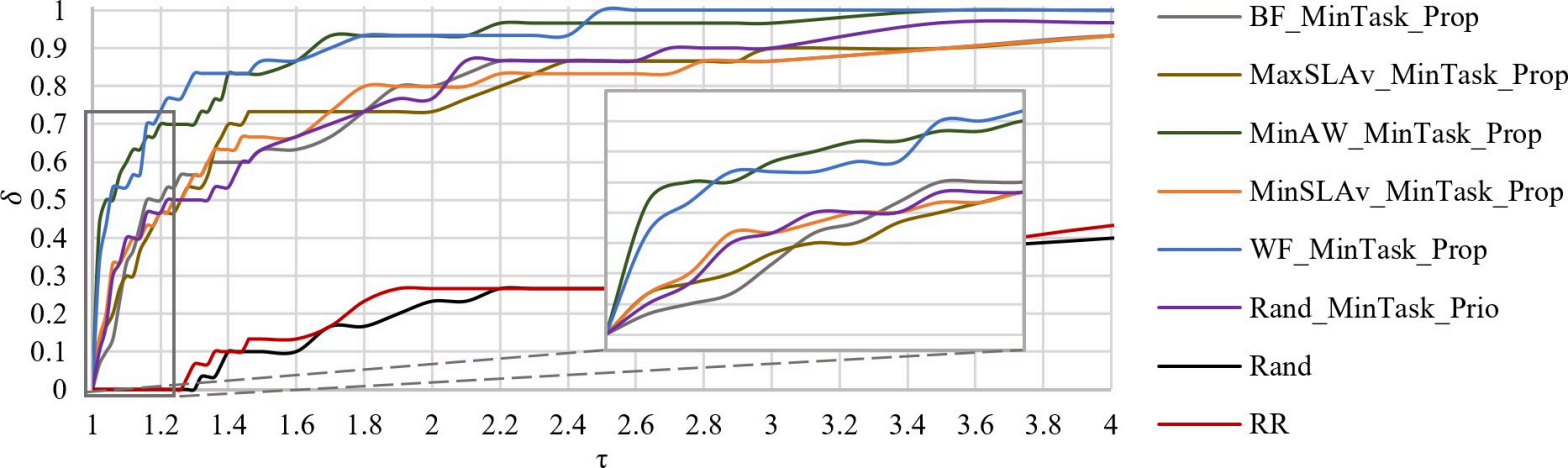
Performance profile of *SLAv*.

Fig 11 shows the *PP* of *SLAv* in the interval τ = [1, 4]. WF_MinTask_Prop generastes all solutions in the interval τ = [1, 2.5]. Hence, it does not produce solutions with *SLAv* more than 2.5 worse than the best found. MinAW_MinTask_Prop generates 96.7% solution in the same interval. Both strategies have similar performance.

**Fig 12:**
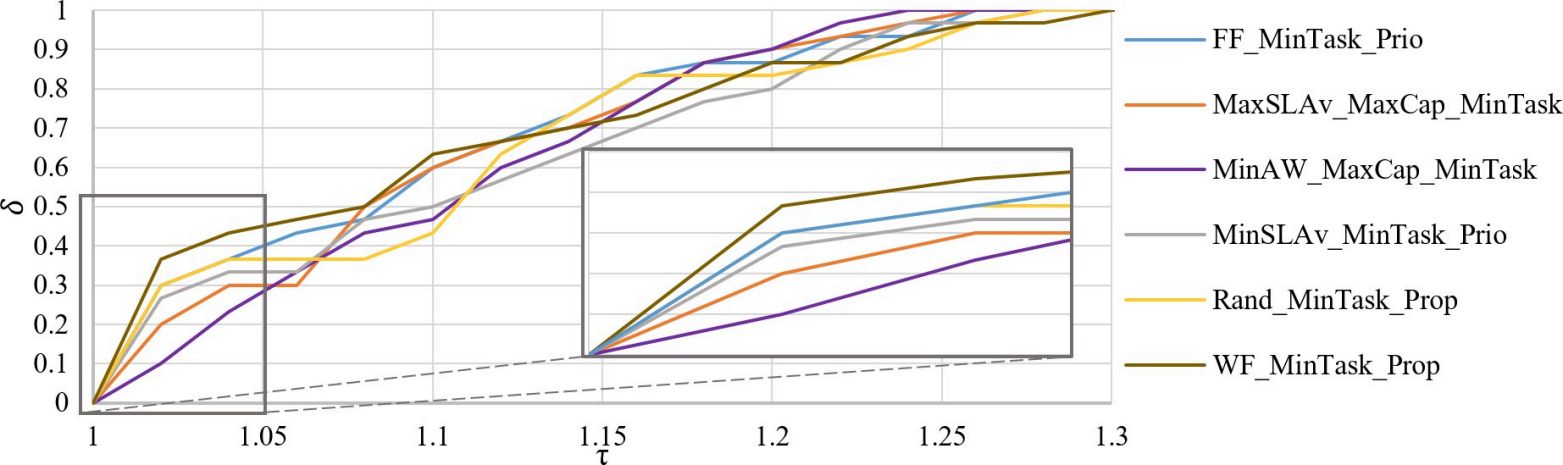
Performance profile of *C*_*max*_.

Fig 12 presents *PP* of *C*_*max*_ in the interval τ = [1.0, 1.3]. WF_MinTask_Prop provides 67% of its solutions within a factor of 1.13 from the best found. It is followed by FF_MinTask_Prio, with 83% of their solution within a factor of 1.18. MinAW_MaxCap_MinTask strategy generates all the solutions within a factor of 1.24.

**Fig 13:**
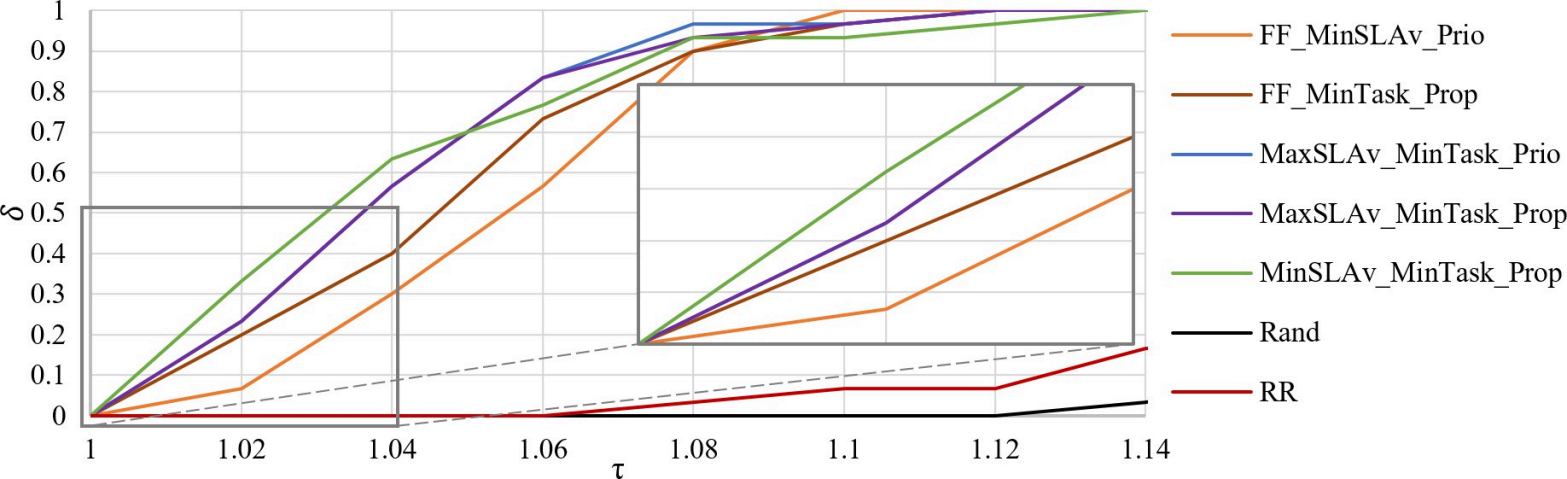
Performance profile of *E*.

Fig 13 shows *PP* of *E* in the interval τ = [1.0, 1.14]. All solutions of FF_MinSLAv_Prio are within a factor of 1.1. It has the best global performance. Two strategies with a high probability of offering good performance are MinSLAv_MinTask_Prop and MaxSLAv_MinTask_Prio. The first strategy provides 70% of its solutions within a factor of 1.05. The second strategy has 95% of solutions below a factor of 1.08.

**Fig 14:**
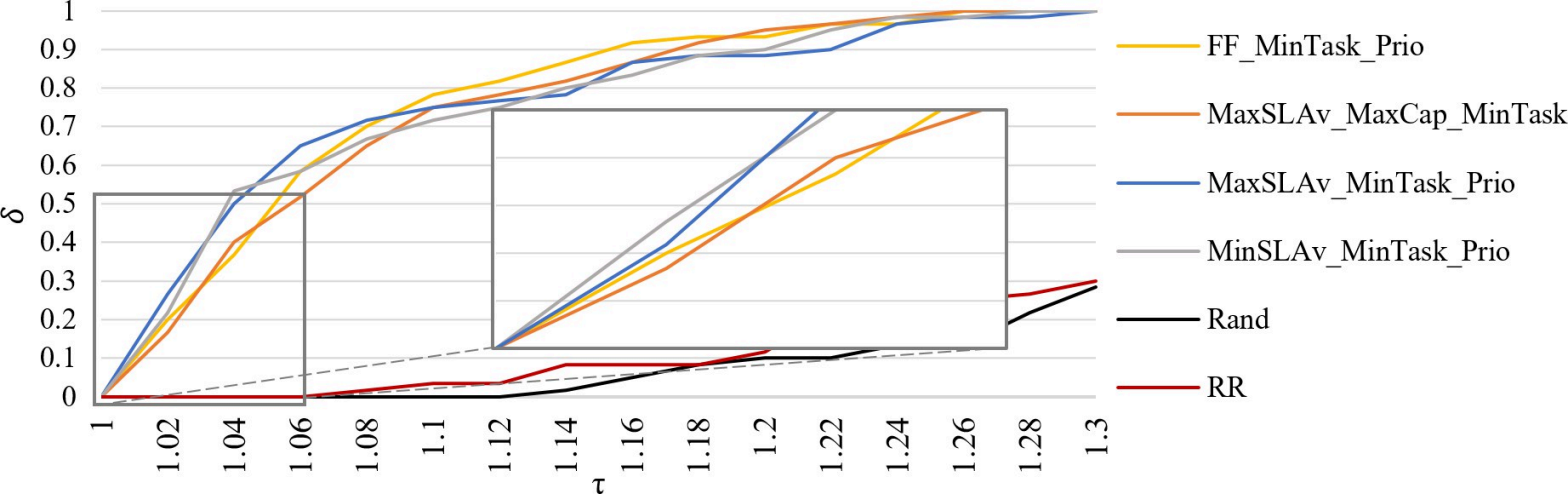
Performance profile of *E* and *C*_*max*_ average.

Fig 14 presents the *PP* of eight strategies according to mean *E* and *C*_*max*_ in the interval τ = [1.0, 1.3]. If we choose a factor of 1.08 from the best result as the scope of our interest, MaxSLAv_MinTask_Prio has a 72% probability of being the winner. FF_MinTask_Prio is the strategy with 92% of solutions within a factor of 1.18. MaxSLAv_MaxCap_MinTask is the strategy with all its solution within a factor of 1.28.

We see that the best strategies include MinTask allocation. Minimizing the number of the running of jobs on the resource reduces possible contention and *SLAv* increasing the assigned speed to each job. Hence, it reduces the energy consumption and job completion time.

### C. Set coverage analysis

Let us analyze Pareto fronts of strategies considering three optimization criteria: *SLA*, *E* and *C*_*max*_.

Two sets of non-dominated solutions are compared using the *SC* metric. The rows of the Table 8 show the values *SC(A*, *B)* for the dominance of strategy *A* over strategy *B*. The columns indicate *SC(B*, *A)*, that is, the dominance of *B* over *A*.

**Table 8:** Set coverage and ranking.

Strategies	BF_MinTask_Prop	FF_MinTask_Prop	MaxSLAv_MinTask_Prop	MinAW_MinTask_Prop	MinSLAv_MinTask_Prop	WF_MinTask_Prop	FF_MinTask_Prio	MinAW_MinTask_Prio	WF_MinTask_Prio	Average best	Rank best	Average all	Rank all
BF_MinTask_Prop	1	0.233	0.200	0.067	0.267	0.067	0.433	0.300	0.267	0.315	**3**	0.544	**1**
FF_MinTask_Prop	0.033	1	0.100	0.067	0.167	0.067	0.200	0.167	0.167	0.219	7	0.432	7
MaxSLAv_MinTask_Prop	0.000	0.200	1	0.133	0.200	0.033	0.300	0.300	0.167	0.259	4	0.464	**3**
MinAW_MinTask_Prop	0.100	0.233	0.267	1	0.200	0.167	0.300	0.400	0.267	0.326	**2**	0.462	4
MinSLAv_MinTask_Prop	0.100	0.133	0.300	0.033	1	0.000	0.233	0.233	0.200	0.248	5	0.450	6
WF_MinTask_Prop	0.100	0.300	0.300	0.200	0.167	1	0.300	0.367	0.267	0.333	**1**	0.516	**2**
FF_MinTask_Prio	0.100	0.067	0.133	0.100	0.133	0.000	1	0.133	0.067	0.193	8	0.413	8
MinAW_MinTask_Prio	0.100	0.033	0.067	0.067	0.067	0.033	0.167	1	0.067	0.178	9	0.402	9
WF_MinTask_Prio	0.067	0.133	0.100	0.033	0.100	0.033	0.267	0.333	1	0.230	6	0.457	5
Average best	0.178	0.259	0.274	0.189	0.256	0.156	0.356	0.359	0.274				
Rank	**2**	5	6	**3**	4	**1**	7	8	6				
Average all	0.022	0.035	0.035	0.023	0.033	0.020	0.049	0.050	0.027				
Rank	**2**	6	6	**3**	5	**1**	7	8	4				

The column “Average best” shows *SC* of row *A* over each of the best strategies. The column “Average all” shows *SC* of row *A* over all studied strategies. The rankings “Rank best” and “Rank all” are based on the average dominance over best and all studied strategies, respectively.

Similarly, the last four rows show average dominance *B* over *A*, average dominance *B*, and two ranks in each column, respectively. The higher ranking implies that the front is better. The most significant values are highlighted in bold and red.

According to the *SC* metric, the strategies with the best compromise between *SLAv*, *E* and *C*_*max*_ are WF_MinTask_Prop, BF_MinTask_Prop, and MaxSLAv_MinTask_Prop considering all strategies, and WF_MinTask_Prop, BF_MinTask_Prop, and MinAW_MinTask_Prop according to the best strategies.

WF is trying to allocate jobs with more capacity left to avoid resource contention. WF_MinTask_Prop improves the non-dominated fronts of other strategies in the range of 10–36.7%, with 33.3% for the best strategies and 51.6% for all strategies, on average, occupying the first and the second ranks, respectively. We see that WF_MinTask_Prop can provide solutions with better quality, on three objectives, with respect to other strategies.

*SC*(*A*, *WF_MinTask_Prop*) displays that strategy is dominated by other strategies with a maximum of 16.7%. These ranges show that other strategies have a better performance than WF_MinTask_Prop. In general, a desired strategy exhibits the behavior of WF_MinTask_Prop. *SC(A*, *RR)* and *SC(A*, *Rand)* are equal to 1, hence, all the strategies dominate RR and Rand. Moreover, *SC(RR*, *B)* and *SC(Rand*, *B)* are zero, so any solution of Rand and RR is dominated by other strategies. We see that similar to the performance profile analysis, best strategies include MinTask allocation. Moreover, the MinTask and *Prop* provide the four best strategies in both ranks.

## VIII. Conclusion

Consolidation of services is a key technology to reduce resource overprovisioning and energy consumption. However, when multiple jobs and processes run on a multicore CPU, they can compete for shared resources such as caches, memory controllers, memory buses, prefetching hardware, disks, networking, etc. This resource contention can invoke performance degradation defeating the benefits of the consolidation, leading to QoS violation and increasing energy consumption.

In this paper, we consider potentially damaging effects of job consolidation. We propose a novel method of consolidation based on the job concentration paradigm that avoids allocation of jobs of the same type on shared resources. It reduces resource contention and makes job placement more efficient with the energy-utilization tradeoff.

We present online job allocation heuristics for heterogeneous infrastructures as a multilevel variant of the dynamic bin-packing problem considering container selection, capacity distribution, and contention-aware allocation. We study eighty-six scheduling heuristics. We distinguish them depending on the type and amount of information they use for allocation. We provide their comprehensive analysis on a real workload.

The results show that proposed allocation strategies carefully guided by the energy, performance, and QoS provide a reduction of 21.73–43.44%, 44.06–92.11%, and 16.38–24.17% of these criteria, respectively, with respect to known strategies used in the literature.

However, further study is required to assess allocation strategies on bigger variety of infrastructures, processors, and containers. It is important to analyze network-intensive, disk-intensive, etc. job types, combining their execution over different scenarios.

## IX. Appendix

**Table A1:** Notation.

Notation	Description
*CI*	CPU intensive jobs
*MI*	Memory intensive jobs
*BI*	Bandwidth intensive jobs
*RC*	Required Capacity model
*Prop*	Full Proportional capacity model
*Prio*	Full Priority capacity model
*SC*	Set Coverage metric
*PP*	Performance Profile
*P* = {*p*_1_, *p*_2_,…,*p*_*M*_}	*M* servers or processors.
*Q*_*k*_	Maximum processing capacity of *p*_*k*_ in Millions of Instructions Per Second (MIPS)
*m*_*k*_	Number of containers running in *p*_*k*_
$C_{k}=\{c_{1}^{k},c_{2}^{k},\ldots ,c_{m_{k}}^{k}\}$	Containers running in *p*_*k*_
$q_{i}^{k}$	Processing capacity of container $c_{i}^{k}$ (CPU quota)
*m*	The total number of containers running in the infrastructure is denoted by $m=\sum_{k=1}^{M}m_{k}$
$J(c_{i}^{k})$	Subset of jobs running in the container $c_{i}^{k}$
$\alpha (c_{i}^{k})$	Number of SLA violations in the container $c_{i}^{k}$
*J* = {*j*_1_, *j*_2_,…,*j*_*n*_}	Set of *n* independent jobs
*r*_*j*_	Release time of *j*_*j*_
*s*_*j*_	Minimum required processing speed of *j*_*j*_
*w*_*j*_	The total amount of work of *j*_*j*_ in millions of instructions
*ρ*_*j*_	Job type of *j*_*j*_
*o*_*j*_	Priority of *j*_*j*_
$s_{j}^{\prime }(t)$	Processing speed of *j*_*j*_ at time *t*
*C*_*max*_	Maximum finishing time (completion time or makespan) of all jobs
*E*	Total energy consumption
*SLAv*	Total number of violations of the service level agreements
*E*^*op*^(*t*)*dt*	Energy consumption of the infrastructure at time *t*
$e_{k}^{proc}(t)$	Energy consumption of the processor *k* at time *t*
*o*(*t*)	If the processor is on at time *t* then *o*(*t*) = 1, otherwise *o*(*t*) = 0
$e_{k}^{idle}$	A constant for idle power or base power of processor *k*
$e_{k}^{used}(t)$	Dynamic energy consumption of processor *k* at time *t*
$e_{k}^{max}$	A constant for the maximum energy consumption of the processor *k* at full capacity
*F*_*k*_(*t*)	Portion of the power consumed by different types of jobs
$g_{k}\left(\varphi_{CI}^{k}(t)\right)$	Fraction of the total energy consumption introduced by job type combinations
$\varphi_{CI}^{k}(t)$	Concentration (proportion) of the job *CI* (quantity of job utilization in a processor utilization) at time *t*
$f_{d}^{k}\left(U_{d}^{k}(t)\right)$	Fraction of energy consumption of a given job type *d*
$U_{d}^{k}(t)$	Utilization of all jobs of type *d* at time *t*
*v*_*j*_	Percentage of $q_{i}^{k}$ assigned to the job *j*_*j*_
${factor}_{c_{i}^{k}}$	Percentage of $q_{i}^{k}$ assigned to the job *j*_*j*_ considering the priority of the job
*δ*(*τ*)	Piecewise constant function
*SC*(*A*, *B*)	Dominance of strategy *A* over strategy *B*

## Supporting information

S1 Fig Time, *SLA* violations, C_max_ and E for all the strategies. (XLSX)

S2 Fig *SLA* violations performance profile. (XLSX)

S3 Fig C_max_ performance profile. (XLSX)

S4 Fig E performance profile. (XLSX)

S5 Fig Performance profile average of E and C_max_. (XLSX)

S1 Table Degradation and ranking. (XLSX)

S2 Table Set coverage and ranking. (XLSX)

S1 File Author bios. (DOCX)
