# Supplementary material for: Dynamic performance–Energy tradeoff consolidation with contention-aware resource provisioning in containerized clouds
Source: PLoS One. 2022 Jan 20;17(1):e0261856. doi: 10.1371/journal.pone.0261856 (PMC8775309; doi:10.1371/journal.pone.0261856)
Supplement: S1 File — (DOCX) [file pone.0261856.s008.docx]

**
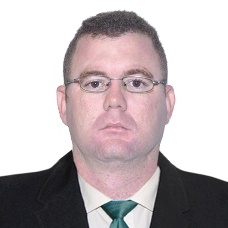
REWER M. CANOSA REYES** received his Bachelor’s degree in Computer Science from the University of Cienfuegos, Cuba in 2007, the Master’s degree in 2018 from CICESE Research Center in Computer Science. His main interests include cloud computing, virtualization, DevOps, containerization, distributed computing, and scheduling.

**
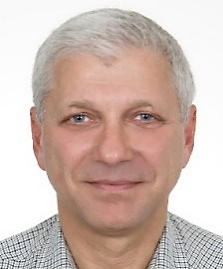
ANDREI TCHERNYKH** received a Ph.D. degree from the Institute of Precise Mechanics and Computer Technology of the Russian Academy of Sciences, Russia in 1986. He is currently a full professor in the Computer Science Department at CICESE Research Center, Ensenada, Baja California, Mexico, and Adjunct Professor at Institute for System Programming of the RAS, Russia. He is head of the Parallel Computing Laboratory at CICESE and Laboratory of Problem-Oriented Cloud Computing at South Ural State University, Russia. He is a member of the National System of Researchers of Mexico (SNI), Level II, and leads several national and international research projects. His main interests include resource optimization technique, adaptive resource provisioning, multi-objective optimization, computational intelligence, incomplete information processing, cloud computing, and security. <https://usuario.cicese.mx/~chernykh>

**
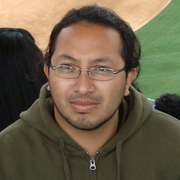
JORGE M. CORTÉS-MENDOZA** received his Bachelor’s degree in Computer Science from the Autonomous University of Puebla (Benemérita Universidad Autónoma de Puebla, México) in 2008, the Master’s degree in 2011 and the Ph.D. degree in 2018 from CICESE Research Center in Computer Science. He is a member of the National System of Researchers of Mexico (SNI) since 2020. His main interests include cloud computing, load balancing, distributed computing, and scheduling.


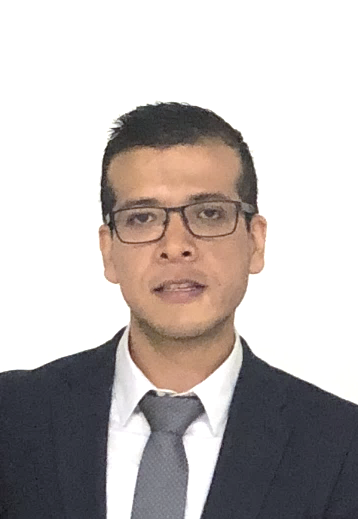
**BERNARDO PULIDO-GAYTAN** received a Bachelor's degree in Engineering in Computer Science from the National Polytechnic Institute, Mexico in 2017, and a Master's degree in Computer Science from CICESE Research Center in 2019. Currently, he is studying his Ph.D. in Computer Science at CICESE Research Center in the optimization of privacy-preserving machine learning cognitive models in cloud environments via homomorphic encryption. His main interests include computational intelligence, multi-objective optimization, scheduling, load balancing, and resource allocation on distributed systems to solve complex optimization problems on cloud computing, including energy consumption and security.

**
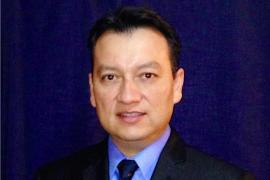
RAUL RIVERA-ODRIGUEZ** received the degree in Electronics Engineering from the Technological Institute of Sonora, Sonora, Mexico, and the degree of Master of Science in Electronics and Telecommunications from the Center for Scientific Research and Higher Education of Ensenada, Baja California (CICESE) in 1994 and 1997, respectively. He received a Ph.D. degree from the Autonomous University of Baja California (UABC), Baja California, Mexico. He is a member of the National System of Researchers of Mexico (SNI), Level I. He contributed to the development of the Infrastructure of the National Research Network for Education in Mexico as President of the CUDI Networks Committee. He is currently Director of the Telematics Directorate of the CICESE in Ensenada, Mexico. His research interests include network management systems, QoS in IP networks, signal processing for wireless communications, multimedia communications, communication networks, cross-layered design, and coding theory.

**
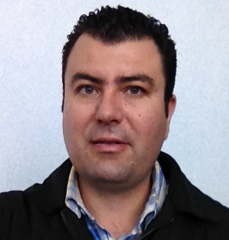
JOSE E. LOZANO-RIZK** received his Bachelor’s degree in Computer Engineering from the Autonomous University of Baja California (UABC, Mexico) in 2003. In 2007 received the Master's degree in Computer Engineering and in 2019 the Ph.D. degree in Computer Science from UABC. He currently works in the Computer Department at CICESE Research Center in the Telematics Division. His research interests are high-performance computing (HPC), Big Data, parallel computing, distributed systems and communications networks for HPC.

**
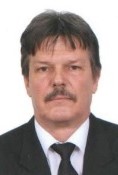
EDUARDO R. CONCEPCION MORALES** received his Ph.D. degree in Computer Science from the University of the Basque Country, Spain, in 2010. He is a professor at the Department of Informatics at the University of Cienfuegos, Cuba; and an invited professor at the Metropolitan University of Ecuador, Quito. He has published more than 30 journal and conference papers. His main interests include cloud computing and scheduling.

**
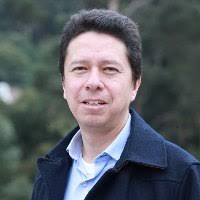
HAROLD ENRIQUE CASTRO BARRERA** graduated as Computing and System Engineer at Universidad de los Andes in Bogota, Colombia. He got a D.E.A (MSc) from the Institut National Polytechnique de Grenoble (INPG), in Grenoble, France and since 1995 he holds a Ph.D. in computer science from INPG also. Associate professor at the Computing and Systems Department at Universidad de los Andes. He is the director of the COMIT (Communications and Information Technology) research group which main research focus are distributed systems and High Performance Computing (HPC) Systems. Dr. Castro personally leads institutional and national cloud and HPC initiatives, and he was national coordinator for the establishment of a grid HPC platform between Europe and Latina America. His interest areas are: HPC, distributed systems and cloud computing.

**
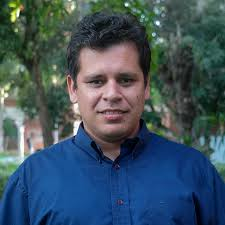
CARLOS J. BARRIOS-HERNANDEZ** received his PhD. In Computer Science from the Université de Nice-Sophia Antipolis in France and the master degree in applied mathematics and informatics at National Institute of Applied Mathematics and Informatics of Grenoble from University of Grenoble-Alpes, same in France. Prof. Barrios currently is associate professor at Universidad Industrial de Santander in Bucaramanga, Colombia and director of the High Performance and Scientific Computing Center of the same University. He is ACM and IEEE Computer Society Senior Member and he collaborate and leads different worldwide projects related with HPC and Advanced Computing. Precisely, his research interests include advanced and high performance computing, large scale systems and soustainable computer architecture. More information at: <http://orcid.org/0000-0002-3227-8651>.

**
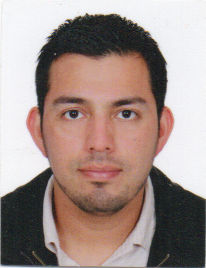
FAVIO MEDRANO-JAIMES** received his Bachelor's degree in Computer Engineering in 2004 and a Master's degree in 2014 from the Autonomous University of Baja California. He works as a technical specialist in scientific computing for the Computing Department in CICESE Research center. Also, he is a member of the Mexican Supercomputing Network (REDMEXSU). His areas of expertise are software engineering, parallel computing, and Big Data solutions.

**
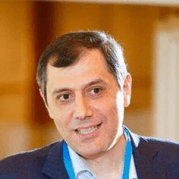
ARUTYUN AVETISYAN** received his master's degree in Applied Mathematics from Yerevan State University in 1993. He received his Ph.D. in computer science in 2012 from the Institute for System Programming of RAS. Since 2015, he is the director of the Institute for System Programming of the Russian Academy of Sciences. He is the head of Samsung Laboratory in the Institute for System Programming of the RAS and the head of the NVIDIA Research Center in the Institute for System Programming of the RAS. His interests are Program Analysis and Transformation, Software Security and, Parallel and Distributed Computing.

**
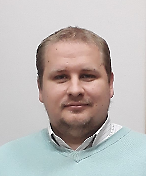
MIKHAIL BABENKO** graduated from Stavropol State University (SSU) in 2007 with a degree in mathematics. Received a Ph.D. degree in mathematics from SSU in 2011. He works as an assistant professor in the Department of Applied Mathematics and Mathematical Modeling since 2012. He is an author of over 63 publications and 5 patents. His research interests include cloud computing, high-performance computing, residue number systems, neural networks, cryptography.

**
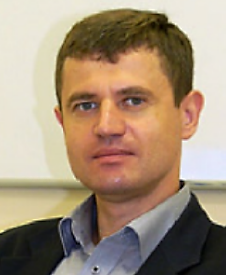
ALEXANDER YU DROZDOV** received his M.Sc. degree in mathematics from the Moscow State University, Russia in 1988. He is currently a Full Professor at The Moscow Institute of Physics and Technology, Russia, and the head of the laboratory of design and modeling of special-purpose computer systems. His research interests are in the fields of research and development of new high-performance architectures and embedded computing systems, embedded control systems, together with the development of tools, embedded and system software.
